# Systemic Perioperative Antimicrobial Prophylaxis for Penile Prosthesis Implantation: A Systematic Review

**DOI:** 10.3390/antibiotics15100958

**Published:** 2026-09-28

**Authors:** Felix Bratosin, Talida Georgiana Cut, Yuthik Nara, Dorin Novacescu

**Affiliations:** 1 Division of Infectious Diseases, Department of Internal Medicine, McGovern Medical School, UTHealth Houston, Houston, TX 77030, USA; felix.bratosin@uth.tmc.edu; 2 Department XIII, Discipline of Infectious Diseases, Victor Babes University of Medicine and Pharmacy Timisoara, 300041 Timisoara, Romania; 3 Department II of Microscopic Morphology, Victor Babes University of Medicine and Pharmacy Timisoara, 300041 Timisoara, Romania; yuthiknara@icloud.com; 4 Department of Human Medicine, Bukovinian State Medical University, 2 Teatralna Square, 58002 Chernivtsi, Ukraine; novacescu.dorin@umft.ro

**Keywords:** penile prosthesis, antibiotic prophylaxis, prosthesis-related infections, antifungal agents, antimicrobial stewardship

## Abstract

Background and Objectives: Penile prosthesis infection usually ends in device removal, whereas unnecessarily broad or prolonged prophylaxis causes toxicity and promotes resistance. We assessed whether the systemic perioperative antibacterial or antifungal strategy, dose or duration is associated with infection, infection-related explantation or reoperation or antimicrobial toxicity after primary or revision implantation. Materials and Methods: PubMed, Europe PMC, OpenAlex and ClinicalTrials.gov were searched from inception to 29 August 2026 using two complementary strategies that combined penile-prosthesis terms with antimicrobial-prophylaxis terms and, separately, with infection or explantation terms, without language or date limits, supplemented by related-article expansion and reference-list checking. Comparative primary studies were eligible. Risk of bias was appraised with RoB 2 and ROBINS-I, and random-effects models were fitted where independent cohorts reported compatible data. Results: Of 4947 records, 11 studies were included: one small randomised trial and ten non-randomised studies. The pooled crude frequency of infection or infection-related explantation was 2.50% (95% CI 1.78–3.50; I2 = 89%), a descriptive summary across heterogeneous outcome definitions rather than an estimate of the infection rate under any regimen. Postoperative oral antibiotics were not associated with lower risk (pooled odds ratio 1.16, 95% CI 0.89–1.53). A higher or weight-based gentamicin dose did not reduce infection (pooled odds ratio 0.75, 95% CI 0.26–2.18) but was associated with more acute kidney injury in the single study that reported comparative toxicity. Guideline-defined regimens carried higher adjusted risk in two overlapping cohorts and no difference in two others. Antifungal prophylaxis was associated with lower infection in two overlapping analyses but not in a revision-only cohort, an exploratory finding that has not been independently replicated. An explicit dose was reported by only three studies, a numerical duration by four and the interval between administration and incision by none, which limits comparison between regimens, and all non-randomised studies had serious risk of bias. Conclusions: Current evidence identifies no superior antibacterial regimen and does not support routine prolonged prophylaxis. The exploratory antifungal signal and the preliminary renal safety signal with weight-based gentamicin warrant prospective, standardised multicentre evaluation before either informs routine practice.

## 1. Introduction

Penile prosthesis implantation is an established treatment for erectile dysfunction that is refractory to, or unsuitable for, medical management [1,2,3]. The implant creates a permanent foreign-body surface on which a small bacterial or fungal inoculum can establish a biofilm [4,5], and biofilm has been demonstrated on most clinically uninfected devices removed for mechanical failure [6]. Once infection is clinically established, antimicrobial therapy alone rarely succeeds and management usually requires explantation, salvage washout with reimplantation or delayed reimplantation [7,8], procedures that carry a risk of corporal fibrosis, loss of penile length, higher cost and psychological distress.

This review addresses one component of infection prevention: systemically administered antibacterial and antifungal prophylaxis. Systemic exposure is considered separately from device coatings, dipping solutions, irrigation and skin antisepsis because these interventions differ in mechanism, comparator and evidence base [9,10]. Reported infection frequencies vary widely across eras, populations and devices, and prior syntheses have documented that variation without resolving its causes [11,12]. Because infection commonly ends in explantation, even a small absolute reduction matters to the individual patient; conversely, broader or longer prophylaxis given to nearly every patient may add nephrotoxicity, allergy, *Clostridioides difficile* infection, resistance and cost while providing little benefit [13,14].

Current guidance reflects the absence of randomised evidence. The American Urological Association (AUA) Best Practice Statement recommends an aminoglycoside with a first- or second-generation cephalosporin or vancomycin for prosthetic implantation, discontinued within 24 h [15]; international consultation recommendations endorse similar broad coverage [2]; and general surgical guidelines advise against continuing prophylaxis after wound closure [16,17,18]. Multicentre microbiological studies have shown that a substantial proportion of organisms recovered from infected devices are not covered by guideline-concordant regimens [19] and that fungal organisms, principally *Candida* species, account for a meaningful share of infections [20], which prompted interest in antifungal prophylaxis that a focused systematic review found to rest mainly on indirect evidence [21]. Stewardship has questioned near-universal postoperative oral antibiotics [22,23], and expert commentary has argued for locally informed rather than uniform regimen selection [24].

No existing synthesis answers the practical questions a surgeon faces. Methodologically systematic reviews have addressed risk factors for infection [11], the epidemiology of infectious adverse events [12], antifungal prophylaxis alone, with a search that closed in 2020 [21], and coated versus non-coated devices [25]; the only antibiotic-focused synthesis is a narrative review without protocol, eligibility criteria or risk-of-bias assessment [22], and recent overviews blend systemic, local and device-based measures [23,24]. We therefore asked whether, in adults undergoing primary or revision penile prosthesis implantation, any systemically administered antibacterial or antifungal prophylaxis strategy, defined by agent or regimen, dose, route, oral course, intravenous duration or antifungal addition, is associated with fewer prosthesis infections, less infection-related explantation or reoperation or less antimicrobial toxicity than an alternative strategy. Because dose, duration and timing determine whether prophylaxis is pharmacologically adequate, the completeness with which the primary literature reports them was also examined and is presented as a descriptive finding.

## 2. Materials and Methods

### 2.1. Design, Protocol and Eligibility Criteria

This systematic review of primary comparative clinical evidence is reported according to PRISMA 2020 [26] and, because most comparisons could not be pooled, structured according to the Synthesis Without Meta-analysis (SWiM) guidelines [27]. A protocol covering the question, eligibility criteria, search strategy, extraction fields and appraisal tools was finalised before the eligibility set was locked, and the review was prospectively registered on the Open Science Framework (OSF) registry before outcome data were extracted (registration DOI: https://osf.io/6w2fr). The review was not registered in PROSPERO; because PROSPERO accepts only reviews that have not progressed beyond data extraction, retrospective registration is no longer possible, and the publicly accessible, time-stamped OSF record is reported as the sole registration in accordance with PRISMA 2020 [26] item 24.

Eligible studies enrolled adults undergoing primary or revision implantation of an inflatable or malleable penile prosthesis; evaluated a systemically administered antibacterial or antifungal strategy (peri-incisional regimen or agent, dose, preoperative or postoperative oral course, intravenous duration or antifungal addition); included a concurrent or historical comparison group or reported an adjusted association; and reported prosthesis infection, infection-related explantation, another infectious complication, reoperation attributed to infection or antimicrobial toxicity. Randomised trials, quasi-experimental studies, cohort and case-control studies and comparative administrative-database analyses were eligible without language, date or sample-size limits. Mixed urological prosthetic cohorts were eligible when penile-prosthesis data were reported separately or when the intervention was applied uniformly across the prosthetic population. Reviews, guidelines, editorials, surveys, case reports, uncontrolled series, conference abstracts, protocols, animal, in vitro and pharmacokinetic studies without a clinical infection outcome were excluded, as were studies of non-systemic measures only (coated devices, dipping solutions, irrigation, antisepsis, drains, bundles), studies in which a systemic change was inseparably bundled with non-systemic measures, studies of salvage or treatment of infected implants and descriptive microbiological series of explants. Reports without a readable full text were excluded and counted separately.

### 2.2. Search Strategy

PubMed was searched from inception on 14 July 2026 with two complementary strategies run without limits. Strategy A combined controlled vocabulary and title/abstract terms for penile prostheses (“Penile Prosthesis”[Mesh] OR “penile prosthesis”[tiab] OR “penile prostheses”[tiab] OR “penile implant”[tiab] OR “penile implants”[tiab] OR “penile implantation”[tiab]) with terms for antimicrobial agents and prophylaxis (“Antibiotic Prophylaxis”[Mesh] OR “Anti-Bacterial Agents”[Mesh] OR “Antifungal Agents”[Mesh] OR antibiotic[tiab] OR antibiotics[tiab] OR antimicrobial[tiab] OR antifungal[tiab] OR prophylaxis[tiab]). Strategy B, designed to capture reports that name no antimicrobial term in the title or abstract, combined the same population terms (plus “Penile Implantation”[Mesh]) with infection and explantation terms (“Prosthesis-Related Infections”[Mesh] OR “Surgical Wound Infection”[Mesh] OR infection[tiab] OR infections[tiab] OR infectious[tiab] OR explant[tiab] OR explantation[tiab] OR “device removal”[tiab]). PubMed related-article expansion was seeded on every included report, and the reference lists of the included studies and of the principal prior reviews were checked [11,12,21,22,23,24].

To extend coverage beyond MEDLINE, the same two strategies were translated and run on 29 August 2026 in Europe PMC (title, abstract and keyword fields; all sources, including preprints and patents) and in OpenAlex (title and abstract fields), whose Crossref-based coverage includes journals, conference proceedings and book chapters not indexed in PubMed, and the population terms combined with antimicrobial and infection terms were run in ClinicalTrials.gov. The PubMed strategies were re-run on the same date and returned an identical yield. Records were de-duplicated by PubMed identifier, Digital Object Identifier and normalised title. Embase, Scopus, Web of Science and the Cochrane Library could not be accessed. A separate novelty search combined the review concepts with the terms “systematic review”, “meta-analysis” and “protocol” in PubMed, PROSPERO, the Cochrane Library and the Open Science Framework.

### 2.3. Study Selection, Data Extraction and Risk of Bias

Records were screened based on title and abstract against the eligibility criteria; any record leaving real ambiguity about a systemic antimicrobial comparison, or any pre-1990 record without an abstract whose title suggested a regimen comparison, was carried forward to full text. Full-text assessment applied the criteria in a fixed order (population, systemic exposure, comparator or adjusted association, clinical outcome, publication type), and reasons for exclusion were recorded individually. Data were extracted with a piloted form covering study and population characteristics; exposures, comparators, agent, dose, duration and timing; and event counts, effect estimates, confidence intervals, *p* values, follow-up and adverse events. Values were transcribed from the source report or its verified bibliographic record; absent data were recorded as not reported (NR). The completeness of reporting of agent, dose, duration and timing was recorded for each study as a descriptive outcome. For the quantitative syntheses only, an event count was derived from a reported percentage and denominator when the count was not printed, and such values are flagged in the figures. Title-and-abstract screening, full-text adjudication, data extraction and risk-of-bias assessment were performed by one reviewer (F.B.). A second reviewer (T.G.C.), working independently of the first, then verified every full-text eligibility decision, every extracted value and every risk-of-bias judgement against the source records; discrepancies were resolved by discussion, with the fourth author (D.N.) consulted when necessary. Title-and-abstract screening was not performed in duplicate. Overlap between reports drawing on the same multicentre dataset was mapped explicitly.

Risk of bias was assessed with RoB 2 for the randomised trial [28] and ROBINS-I for non-randomised studies [29]. Confounding was judged moderate when a multivariable model adjusted for diabetes, revision status and prior device infection, and serious when the prophylaxis comparison was unadjusted; outcome measurement was judged serious when infection was inferred from an administrative explantation code; and selection of the reported result was judged serious when a prophylaxis variable was analysed but its estimate was not reported.

### 2.4. Synthesis Methods

Meta-analysis across the whole evidence base was not appropriate because exposures (agent, dose, oral course, intravenous duration, antifungal addition), outcomes (clinically defined infection, time to infection, administrative explantation) and metrics (unadjusted proportions, odds ratios and hazard ratios with different adjustment sets) were not commensurable, and because two of the largest analyses draw on the same collaborative dataset. Quantitative synthesis was restricted to the two domains in which independent cohorts reported compatible crude event data (postoperative oral antibiotics versus none; higher or weight-based versus standard gentamicin dose) and to the overall frequency of infection or infection-related explantation. DerSimonian–Laird random-effects models were fitted on the logit scale for proportions and on the log-odds scale for comparisons, with a 0.5 continuity correction for empty cells, and heterogeneity was quantified with I2 and Cochran Q [30]. Following SWiM, the remaining studies were grouped by exposure domain, and their adjusted estimates were displayed side by side without pooling. Certainty was described narratively; GRADE was not applied.

## 3. Results

### 3.1. Study Selection

The study-selection process is summarised in Figure 1. The two PubMed strategies returned 996 unique records and related-article expansion a further 675, of which 498 were new. Europe PMC returned 1063 records, OpenAlex 2167 and ClinicalTrials.gov 46, giving 4947 records in total, of which 2143 were duplicates. After screening 2804 records, 2782 were excluded based on title and abstract. This high exclusion rate was expected, because both search strategies were deliberately sensitive: Strategy B and the supplementary databases retrieved records that mentioned infection or explantation without any antimicrobial term, and the two strategies together captured much of the general literature on penile prosthesis surgery. Most excluded records concerned surgical technique, device design, patient satisfaction or mechanical outcomes without an antimicrobial comparison; non-comparative case series and case reports; reviews, guidelines, editorials and surveys; non-systemic measures such as coated devices, dipping solutions, irrigation or antisepsis; the treatment or microbiology of already-infected implants; or other urological prostheses and non-prosthetic procedures. The remaining 22 reports were assessed in full text, and 11 were excluded: a practice-pattern survey, a cohort with a uniform regimen and no systemic contrast, two cohorts of already-infected devices, two commentaries, two reports without retrievable full text, two studies bundling a systemic change with antiseptic or protocol measures and one comparison of outpatient with inpatient care. Eleven studies were included [31,32,33,34,35,36,37,38,39,40,41]: one randomised trial [31] and ten non-randomised studies [32,33,34,35,36,37,38,39,40,41].

Of the 1310 records retrieved only by the supplementary databases, none was eligible. The 66 Europe PMC records comprised 18 patents, 19 preprints, 5 conference abstracts, 5 reviews or surveys and 19 case reports or studies of other exposures. The 1198 OpenAlex records comprised 323 conference abstracts, book chapters, theses or other non-full-length items; 132 reviews, editorials, letters or surveys; 586 studies of other exposures or non-comparative series; 107 studies of non-systemic measures; and 50 studies of infected-implant treatment or explant microbiology. The 46 registrations included one ongoing randomised trial of postoperative oral antibiotics without results [42] and one trial of a non-systemic irrigation solution. The novelty search identified no completed systematic review or meta-analysis addressing the review question; the closest syntheses address antifungal prophylaxis alone [21], antibiotics in a narrative format [22], risk factors [11], infectious adverse events [12] or coated devices [25].

### 3.2. Overview of the Included Studies

The characteristics of the 11 included studies are summarised in Table 1. All were published between 1996 and 2025, ten were retrospective, and sample sizes ranged from 20 to 26,574. Ten studies were conducted wholly or partly in the United States, four multicentre cohorts also included European, Korean, Saudi or Australian centres [36,38,39,41], and one was a single-centre Spanish cohort [37]. Two studies were national administrative analyses [33,40]. The primary-implant [38] and combined primary-and-revision [41] analyses were drawn from the same international collaborative network, the revision cohort [39] used participating centres from that network and the diabetic cohort [36] was assembled from overlapping institutions during an earlier period; samples should therefore not be summed.

### 3.3. Systemic Antimicrobial Exposures and Comparators

Exposures, comparators and pharmacological detail are summarised in Table 2. Three studies compared gentamicin dosing strategies [32,34,38], three evaluated postoperative oral antibiotics [33,35,37], one evaluated the duration of intravenous prophylaxis [41], four compared guideline-defined with alternative regimens [36,38,39,40], one trial compared an oral with a parenteral regimen [31] and three multicentre reports evaluated antifungal prophylaxis [38,39,41]. An explicit antibacterial dose was retrievable for three studies, all concerning gentamicin, and a numerical duration for four; only one study reported the length of the postoperative oral course [33]. No study reported the interval between administration and incision, intraoperative redosing, renal dose adjustment or allergy substitution. This incompleteness, discussed further in Section 4.4, constrains every comparison that follows, because regimens that differ in name may not differ in delivered exposure and vice versa.

### 3.4. Outcomes of the Included Studies

The principal comparative outcomes are reported in Table 3, and the adjusted estimates for every study are shown in Figure 2. Postoperative oral antibiotics were not associated with lower 90-day explantation in the national claims cohort, in which each additional treatment day carried an adjusted odds ratio of 1.03 (95% CI 1.002–1.05) [33], nor with fewer infections or explantations in the risk-stratified cohort [35] or the international cohort [41]. The Spanish cohort reported a lower frequency of discharge ciprofloxacin among infected than uninfected patients on univariable analysis only [37]. Guideline-defined prophylaxis was associated with higher adjusted odds of infection (OR 2.8, 95% CI 1.1–7.3) and explantation (OR 3.6, 95% CI 1.4–9.1) in diabetic men [36], and vancomycin plus gentamicin with a higher adjusted infection hazard (HR 2.7, 95% CI 1.4–5.4) in the primary-implant cohort [38], whereas the administrative cohort found no adjusted association [40], and the revision cohort reported no association without publishing estimates [39]. Antifungal prophylaxis was associated with lower infection in the primary-implant (HR 0.08, 95% CI 0.03–0.19) [38] and combined (OR 0.22, CI not reported) [41] cohorts but not in the revision-only cohort [39]. Weight-based gentamicin was associated with more acute kidney injury than standard dosing (15.8% versus 3.0%, *p* = 0.02) without less infection [34]. No included study reported the causative organisms of the infections separately for the compared prophylaxis groups, and the antifungal agent was not specified for the analysed exposure in the three reports that evaluated antifungal prophylaxis, so the microbiological effect of any strategy, including whether antifungal prophylaxis reduced specifically fungal infection, could not be assessed (Table 2).

### 3.5. Quantitative Synthesis

Figure 3 shows the frequency of infection or infection-related explantation in the ten studies that reported or allowed derivation of an overall event count. The pooled frequency was 2.50% (95% CI 1.78–3.50) with high heterogeneity (I2 = 89%, Q = 82.8, df = 9, *p* < 0.001), reflecting outcome definitions that ranged from implant loss to 90-day administrative explantation. Study-level frequencies ranged from 0% in the 20-patient trial to approximately 5% in single-centre cohorts that applied a clinical definition of infection. The pooled value should therefore be read as a descriptive summary of the burden of infection-related events across the evidence base, useful for context and for judging the statistical power of the included comparisons, rather than as an estimate of the infection rate attributable to any regimen or as a benchmark against which individual centres or strategies should be judged.

Figure 4 presents the comparative odds ratios calculated from crude event counts by exposure domain. Postoperative oral antibiotics were not associated with lower risk in two independent cohorts (pooled OR 1.16, 95% CI 0.89–1.53; I2 = 0%) [33,35], and a higher or weight-based gentamicin dose was not associated with less infection (pooled OR 0.75, 95% CI 0.26–2.18; I2 = 0%) [32,34]. The two cohorts comparing guideline-defined with alternative regimens produced crude odds ratios in opposite directions, 3.02 (95% CI 1.33–6.86) [36] and 0.83 (95% CI 0.46–1.49) [38], and were not pooled, because their source networks overlap.

### 3.6. Risk of Bias

Risk-of-bias judgements are shown in Figure 5. The randomised trial raised some concerns because sequence generation and allocation concealment were not described, outcome assessment was not blinded and the clinical outcomes were secondary to a pharmacokinetic objective [31]. All ten non-randomised studies were judged at serious overall risk of bias, most often for confounding, participant selection, classification of heterogeneous regimens, outcome measurement based on administrative codes and selection of the reported result [32,33,34,35,36,37,38,39,40,41]; none was judged at low or critical risk.

### 3.7. Synthesis Across Exposure Domains

Grouped by exposure domain, studies of postoperative oral antibiotics or longer intravenous prophylaxis reported no difference or an association in the direction of harm [33,35,41], with one univariable exception [37]. Comparisons of guideline-defined regimens comprised two associations in the direction of higher risk and two null findings [36,38,39,40]. Gentamicin dose comparisons were null for infection, while weight-based dosing was associated with more acute kidney injury [32,34,38]. Two overlapping analyses reported lower infection with antifungal prophylaxis, and the revision-only report was null after adjustment [38,39,41]. No study compared systemic prophylaxis with no prophylaxis or reported comparative outcomes for *Clostridioides difficile* infection, allergic reaction or resistant-organism selection.

## 4. Discussion

### 4.1. Principal Findings

This review assembled the comparative clinical evidence on systemically administered perioperative antimicrobial prophylaxis for penile prosthesis implantation across four bibliographic sources and a trial registry. No regimen has been shown to be superior, and none has been ruled out, because the eleven eligible studies ask different questions with different exposure definitions and outcome structures. Three findings are nevertheless consistent. First, postoperative oral antibiotics and intravenous prophylaxis beyond 24 h were not associated with lower infection or explantation in any adjusted analysis, and the pooled crude estimate from the two independent cohorts was close to unity with no detectable heterogeneity. Second, a higher or weight-based gentamicin dose was not associated with less infection but was associated with more acute kidney injury in the single study that reported comparative toxicity. Third, perioperative antifungal prophylaxis showed an exploratory association with lower infection risk in two analyses that draw on overlapping cohorts, which was not reproduced in the revision-only cohort. These patterns arise almost entirely from non-randomised data at serious risk of bias, so they can inform priorities for practice and research but cannot establish causal superiority. Underlying all three is a more basic limitation, developed in Section 4.4: an explicit dose was retrievable for only three studies, a numerical duration for four and the interval between administration and incision for none, so every comparison between regimens rests on incompletely characterised exposures.

### 4.2. Comparison with Previous Literature

Earlier syntheses addressed patient and procedural risk factors [11], the epidemiology of infectious adverse events [12], antifungal prophylaxis in isolation [21] or antibiotic-coated devices [25], while the antibiotic-focused publication was narrative and lacked a protocol, eligibility framework or risk-of-bias assessment [22]. None jointly evaluated regimen choice, antifungal addition and prophylaxis duration, and none incorporated the large comparative cohorts published after their searches closed [38,40,41]. The present review adds these cohorts, maps their shared source populations so that apparent replication is not mistaken for independent confirmation and extends the search beyond MEDLINE. The supplementary databases retrieved more than a thousand additional records, largely conference abstracts, book chapters, patents and preprints, but no additional eligible study, which suggests that the comparative literature on this question is concentrated in a small number of MEDLINE-indexed urological journals. Several conference abstracts describing single-institution comparisons of extended postoperative prophylaxis were identified but have not been published as full reports.

The conclusions of the primary-study authors reflect the comparison each dataset could support. Schwartz et al. considered oral ofloxacin compatible with outpatient surgery [31], whereas Xie et al. described only a trend toward lower infection with high-dose gentamicin [32]. Adamsky et al. and Dropkin et al. questioned routine postoperative antibiotics [33,35], and Moore et al. cautioned that weight-based gentamicin could increase acute kidney injury without reducing infection [34]. Rezaee et al. and Barham et al. reported higher infection risk with guideline-defined prophylaxis [36,38], while Palmisano et al. reported an inverse univariable association between discharge ciprofloxacin and early infection that was not carried into the multivariable model [37]. The revision cohort attributed complications principally to partial component exchange [39], Brant et al. documented increasing guideline adherence over time [40] and Abou Chawareb et al. reported lower infection with antifungals but limited utility of oral and prolonged intravenous prophylaxis [41].

### 4.3. Interpretation of the Duration, Regimen and Antifungal Findings

The duration findings align with the evidence from surgery as a whole. The AUA statement recommends discontinuing prophylaxis within the early postoperative period [15], and general surgical guidelines advise that no further dose be given once the incision is closed [16,17,18]. That position rests on randomised evidence across surgical specialties showing no reduction in surgical site infection from continuing prophylaxis after operation [43]. The device-specific cohorts reviewed here point the same way, although residual confounding remains possible, because clinicians may extend therapy preferentially for patients perceived to be at higher risk. The most defensible interpretation is that routine extension lacks demonstrated benefit while a narrowly defined high-risk subgroup has not been adequately tested; the ongoing multicentre randomised trial comparing 24 h of intravenous prophylaxis with an additional six days of oral antibiotics, with an estimated 800 participants and completion expected in 2027, should provide the first direct answer [42].

The antibacterial-regimen literature illustrates why categorical labels are weak proxies for pharmacological coverage. Guideline-defined regimens were designed to provide broad Gram-positive and Gram-negative coverage [2,15], yet microbiological studies have shown incomplete coverage of organisms recovered from infected implants [19]. In the included cohorts, the comparator category often grouped pharmacologically dissimilar combinations, and timing, redosing, renal adjustment and allergy substitution were seldom available, so an association between a named regimen and infection may reflect centre practice, patient selection, local ecology or operative complexity rather than the drugs themselves. Surgeon volume is a case in point: in a state-wide analysis of 14,969 patients, those treated by lower-volume surgeons were roughly twice as likely to require reoperation for infection [44]. The same reasoning underlies evidence that infection risk is strongly shaped by patient and procedural factors [11], European guidance that places stewardship and duration at the centre of urological antimicrobial practice [45] and expert calls for locally informed prophylaxis [24].

The antifungal signal connects a plausible biological target with newer outcome data. *Candida* species constitute a meaningful proportion of organisms recovered from infected devices [20] and are not addressed by standard antibacterial regimens [19]. Experience with fungal periprosthetic joint infection, in which *Candida* predominates and implant retention rarely succeeds, points the same way [46,47]. Whereas the earlier focused review found support for antifungal prophylaxis to be largely indirect [21], the multicentre analyses now provide outcome-based associations [38,41]; however, the two reports overlap substantially, the revision-only cohort showed no association [39], confidence intervals were incompletely reported and confounding by centre practice is plausible. The signal warrants prospective testing rather than a change in routine practice.

### 4.4. Safety, Stewardship and Implications for Practice

The only comparative toxicity study found more acute kidney injury with weight-based gentamicin and no reduction in infection [34], consistent with the known dose- and exposure-related nephrotoxicity of aminoglycosides in surgical prophylaxis [48,49]. That finding rests, however, on nine versus two events in a single retrospective cohort of 124 procedures that also included artificial urinary sphincter surgery and reported no adjusted estimate, so it should be regarded as a preliminary safety signal rather than an established comparative harm; comparative toxicity data for the other regimens do not exist. Harm also accrues from duration, because each additional day of postoperative prophylaxis has been associated with acute kidney injury, *Clostridioides difficile* infection and acquisition of resistant organisms without a reduction in surgical site infection [13,50], and, against the global burden of bacterial resistance [14], discretionary antimicrobial exposure is not negligible. In practice, prophylaxis decisions should incorporate renal function, allergy history, interacting medications, local bacterial and fungal epidemiology, prior device infection and whether surgery is primary or revision, and shared decision-making should acknowledge both the serious consequences of prosthesis infection and the uncertainty surrounding broader or longer exposure.

A further finding is what the primary literature does not report. Dose, duration and timing determine whether a regimen achieves adequate tissue concentration at incision, and they are the variables that general surgical guidance specifies most precisely [16]. In this evidence base, an explicit dose was retrievable for three studies and a numerical duration for four, no study reported the interval between administration and incision and two studies classified regimens entirely from prescription or billing records [33,40]. A literature that cannot state how much of a drug was given, when or for how long cannot support a comparison between regimens, and this limitation is more fundamental than the imprecision of any individual estimate.

### 4.5. Limitations

The evidence has important limitations. Nearly all included studies were non-randomised and at serious overall risk of bias, so confounding by indication, centre, surgeon, comorbidity and operative complexity remains plausible, and the single randomised trial contributed no estimable clinical effect. Exposure definitions were coarse, administrative explantation codes may miss infections managed without removal and include removals for non-infectious reasons, overlap between the major multicentre analyses prevents their concordant antifungal findings from being read as independent replication and one study combined penile and urinary prostheses without a device-specific split. Because dose and duration were reported by so few studies, the dose and duration components of the review question could be examined only in the gentamicin and oral-course comparisons, and the review answers them principally by documenting the absence of data.

The review process also has limitations. Quantitative synthesis was confined to two exposure domains, the pooled frequency of infection-related events summarises heterogeneous outcome definitions and has descriptive rather than inferential meaning and some event counts used in the figures were derived from a reported percentage and denominator. The search combined PubMed, Europe PMC, OpenAlex and ClinicalTrials.gov with related-article expansion and reference-list checking, but Embase, Scopus, Web of Science and the Cochrane Library could not be accessed, and grey literature and conference abstracts were excluded by design. The omission of these databases could have reduced the completeness of the evidence base, most plausibly by missing comparative reports published in European or non-English journals indexed in Embase but not in MEDLINE, or trials captured only by CENTRAL. Several observations limit the likely impact: OpenAlex and Europe PMC index the Crossref, preprint and patent literature that these databases would have contributed and returned more than 1300 additional records without an eligible study; related-article expansion and the reference lists of the included studies and of prior reviews on this topic identified no study absent from the database yield; and the comparative literature on this question appears to be concentrated in a small number of MEDLINE-indexed urological journals. Nevertheless, a small number of additional comparative cohorts cannot be excluded, and because such studies are likely to be small and single-centre, their omission would more plausibly affect the pooled frequency of infection and its heterogeneity than the direction of the comparative findings. Screening, adjudication, extraction and risk-of-bias assessment were completed by one reviewer and then verified by a second reviewer rather than being performed independently in duplicate from the outset, so an error at the title-and-abstract stage, which was not duplicated, could have led to the omission of an eligible study. Reports without readable full text were excluded, selective reporting within primary studies could not be excluded, GRADE was not applied and comparative adverse-event reporting was so sparse that the absence of reported toxicity should not be read as evidence of safety.

### 4.6. Future Research

Future work should prioritise a pragmatic multicentre randomised comparison or a prospectively harmonised target trial emulation in which eligibility, treatment strategies, assignment, follow-up and outcomes are specified in advance and reproduced in observational data [51]. A factorial design could test a precisely specified antibacterial backbone, the addition of an antifungal agent and discontinuation at wound closure versus a short postoperative course, stratified by primary or revision surgery, diabetes, previous device infection, immunosuppression, device type and centre. Exposure recording should include agent, dose, route, timing, redosing, renal adjustment and allergy substitution; outcomes should be centrally adjudicated and reported separately for superficial wound concerns, microbiologically confirmed prosthesis infection, salvage, explantation and reimplantation, with active ascertainment of renal, allergic, drug-interaction and microbiological harms. Because infection is uncommon, collaborative registries with common data standards may be necessary to separate the effect of a drug from the practices of the centres that use it; arthroplasty registries offer a working model [52]. The ongoing trial of postoperative oral antibiotics [42] illustrates that such studies are feasible, and a parallel trial of antifungal addition would address the most promising unresolved signal.

## 5. Conclusions

Comparative evidence on systemic perioperative antimicrobial prophylaxis for penile prosthesis implantation remains observational, heterogeneous and at serious risk of bias. The available studies do not support routine postoperative oral antibiotics or prolonged intravenous prophylaxis and do not identify a superior antibacterial regimen; where independent cohorts could be pooled, neither a postoperative oral course nor a higher gentamicin dose was associated with less infection. The association between perioperative antifungal use and lower infection risk is potentially important but exploratory: it derives from overlapping cohorts, was not reproduced in the revision-only cohort and remains vulnerable to confounding, so it should generate hypotheses rather than change practice. The renal safety signal with weight-based gentamicin, observed in a single small retrospective cohort, is preliminary but supports careful dosing and monitoring pending comparative toxicity data. Standardised prospective multicentre studies that record agent, dose, timing and duration, adjudicate infection outcomes and actively survey for harm are needed before universal prophylaxis recommendations can be justified.

## Figures and Tables

**Figure 1:**
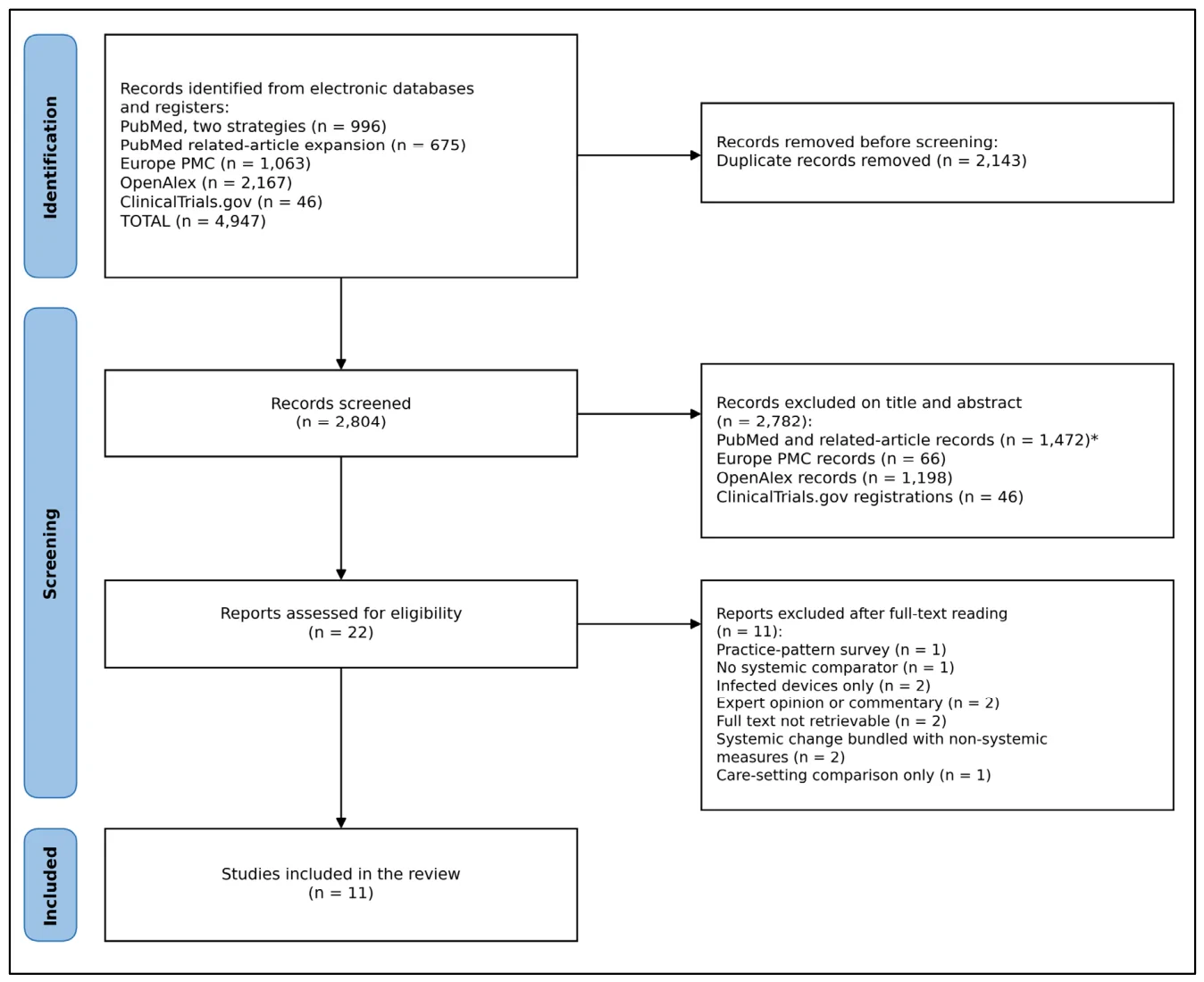
PRISMA 2020 [26] flow diagram of the study-selection process. * Includes one record that could not be retrieved from PubMed. PubMed was searched on 14 July 2026 and re-run on 29 August 2026 with identical yield; Europe PMC, OpenAlex and ClinicalTrials.gov were searched on 29 August 2026.

**Figure 2:**
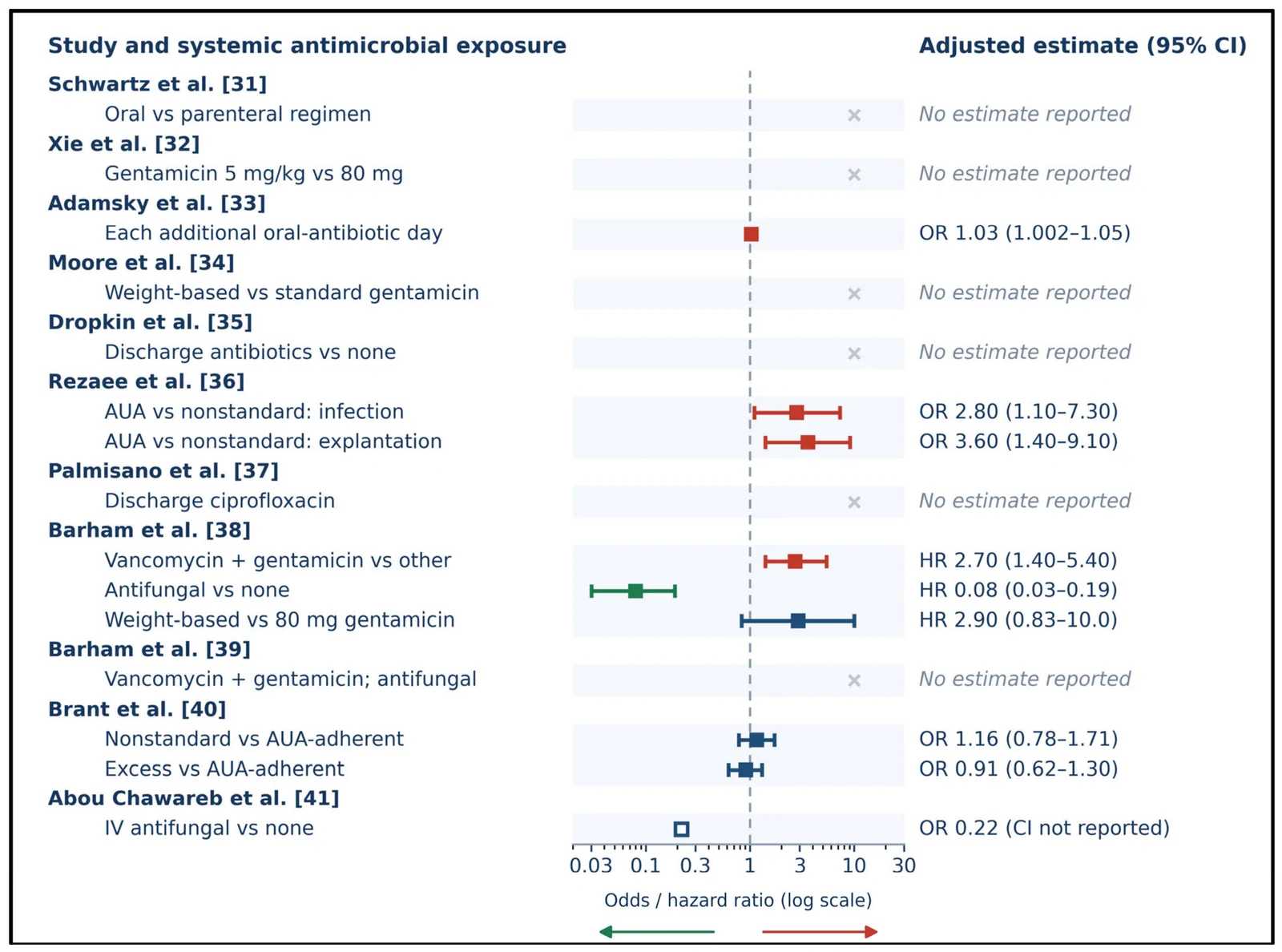
Adjusted effect estimates for systemic antimicrobial exposures in every included study. Estimates are displayed side by side and are deliberately not pooled because they derive from different exposures, outcomes, adjustment sets and, in two cases, overlapping cohorts. Odds ratios and hazard ratios are shown on a common logarithmic axis; studies that reported no adjusted estimate are listed so that completeness of reporting can be judged. The estimate for intravenous antifungal use reported by Abou Chawareb et al. [41] is plotted as an open marker because no confidence interval was reported. Filled squares are point estimates and horizontal bars are 95% confidence intervals; red markers denote estimates whose confidence interval excludes 1 in the direction of higher risk with the exposure, green markers denote estimates whose confidence interval excludes 1 in the direction of lower risk, and blue markers denote estimates whose confidence interval includes 1. Grey crosses denote studies that reported no adjusted estimate. The arrows below the axis indicate that estimates to the left of the dashed line (null value of 1) favour the exposure and estimates to the right of it favour the comparator. Studies shown: Schwartz et al. [31], Xie et al. [32], Adamsky et al. [33], Moore et al. [34], Dropkin et al. [35], Rezaee et al. [36], Palmisano et al. [37], Barham et al. [38], Barham et al. [39], Brant et al. [40] and Abou Chawareb et al. [41].

**Figure 3:**
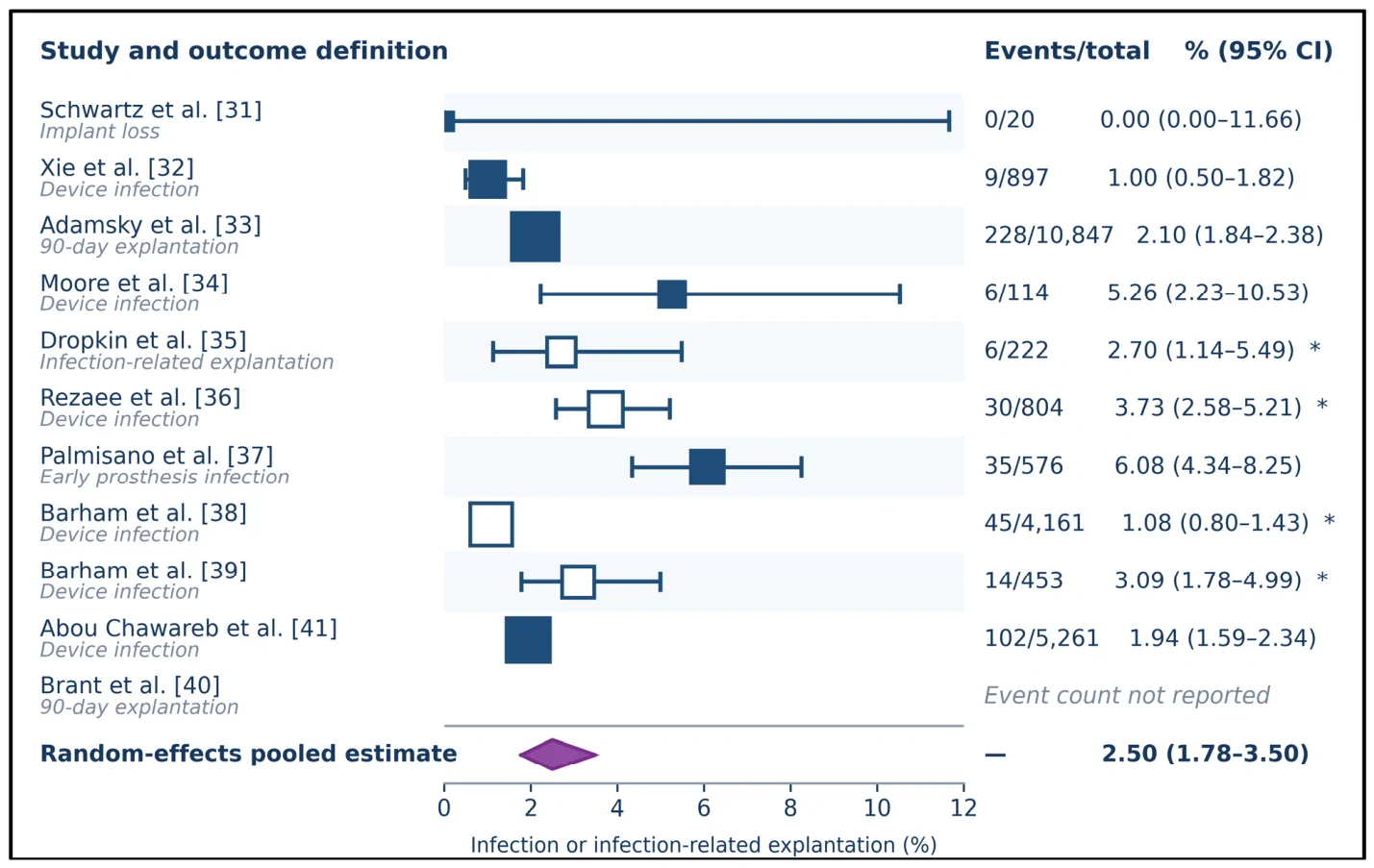
Frequency of infection or infection-related explantation in every included study that reported or allowed derivation of an overall event count, with a DerSimonian–Laird random-effects pooled estimate. Marker area is proportional to sample size, and bars are exact binomial 95% confidence intervals; open markers denote event counts derived from the reported percentage and denominator. Brant et al. [40] is listed for completeness but reported no event count. Asterisks (*) in the right-hand column mark these derived event counts. Squares are study-level point estimates, horizontal bars are exact binomial 95% confidence intervals and the diamond is the random-effects pooled estimate with its 95% confidence interval. Studies shown: Schwartz et al. [31], Xie et al. [32], Adamsky et al. [33], Moore et al. [34], Dropkin et al. [35], Rezaee et al. [36], Palmisano et al. [37], Barham et al. [38], Barham et al. [39], Brant et al. [40] and Abou Chawareb et al. [41].

**Figure 4:**
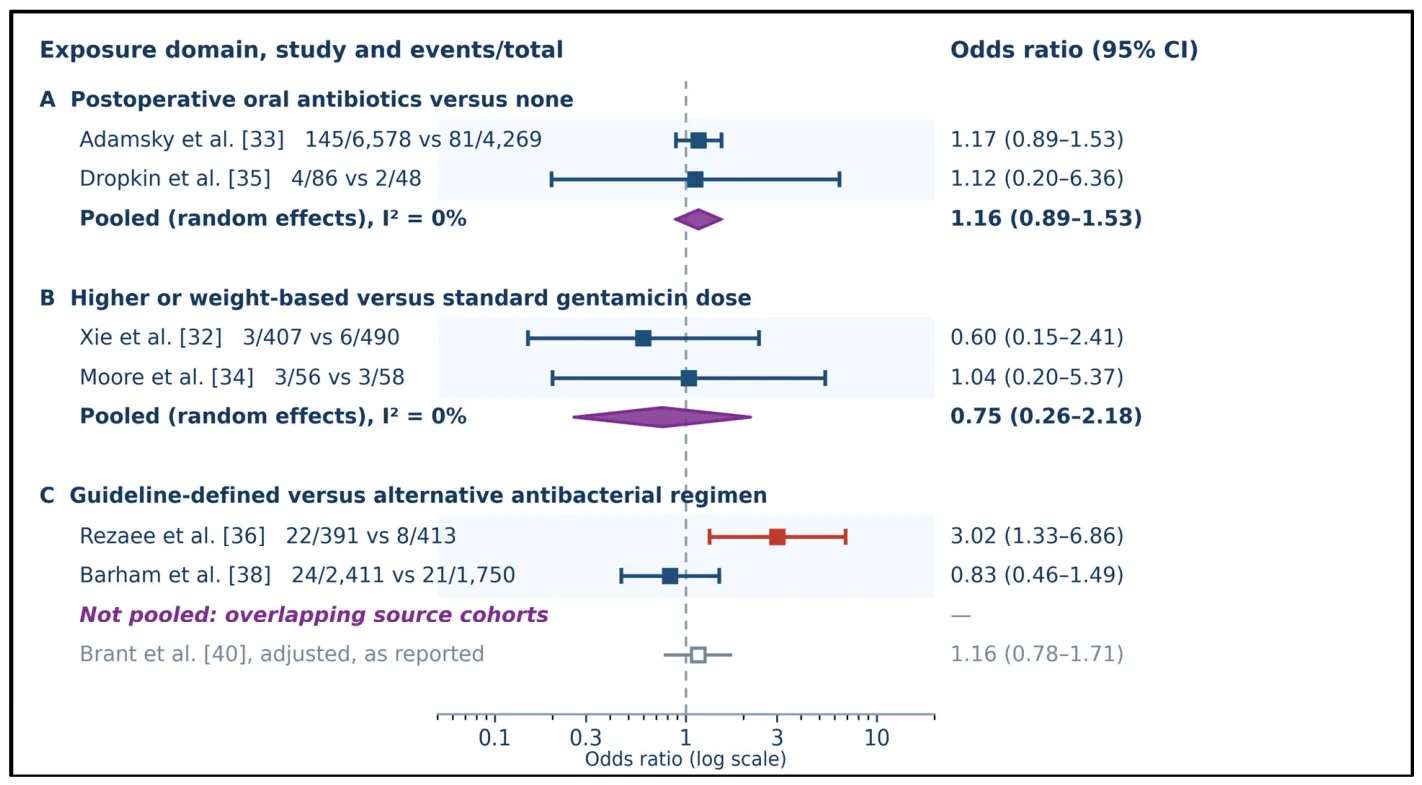
Comparative odds ratios by exposure domain calculated by the review authors from the crude event counts reported by each study. Panels (**A**) and (**B**) show the two domains in which independent cohorts reported compatible data and are accompanied by DerSimonian–Laird random-effects pooled estimates. Panel (**C**) is not pooled, because the two contributing cohorts draw on overlapping collaborative datasets; the adjusted estimate reported by Brant et al. [40] is shown for comparison only. Derived counts may differ by one or two events from the totals in Table 3. Squares are study-level odds ratios, horizontal bars are 95% confidence intervals and diamonds are pooled estimates; the red marker denotes the only estimate whose confidence interval excludes 1, and the open grey marker denotes the adjusted estimate of Brant et al. [40], which was reported by that study rather than calculated by the review authors. Studies shown: Adamsky et al. [33] and Dropkin et al. [35] in panel (**A**); Xie et al. [32] and Moore et al. [34] in panel (**B**); Rezaee et al. [36] and Barham et al. [38] in panel (**C**).

**Figure 5:**
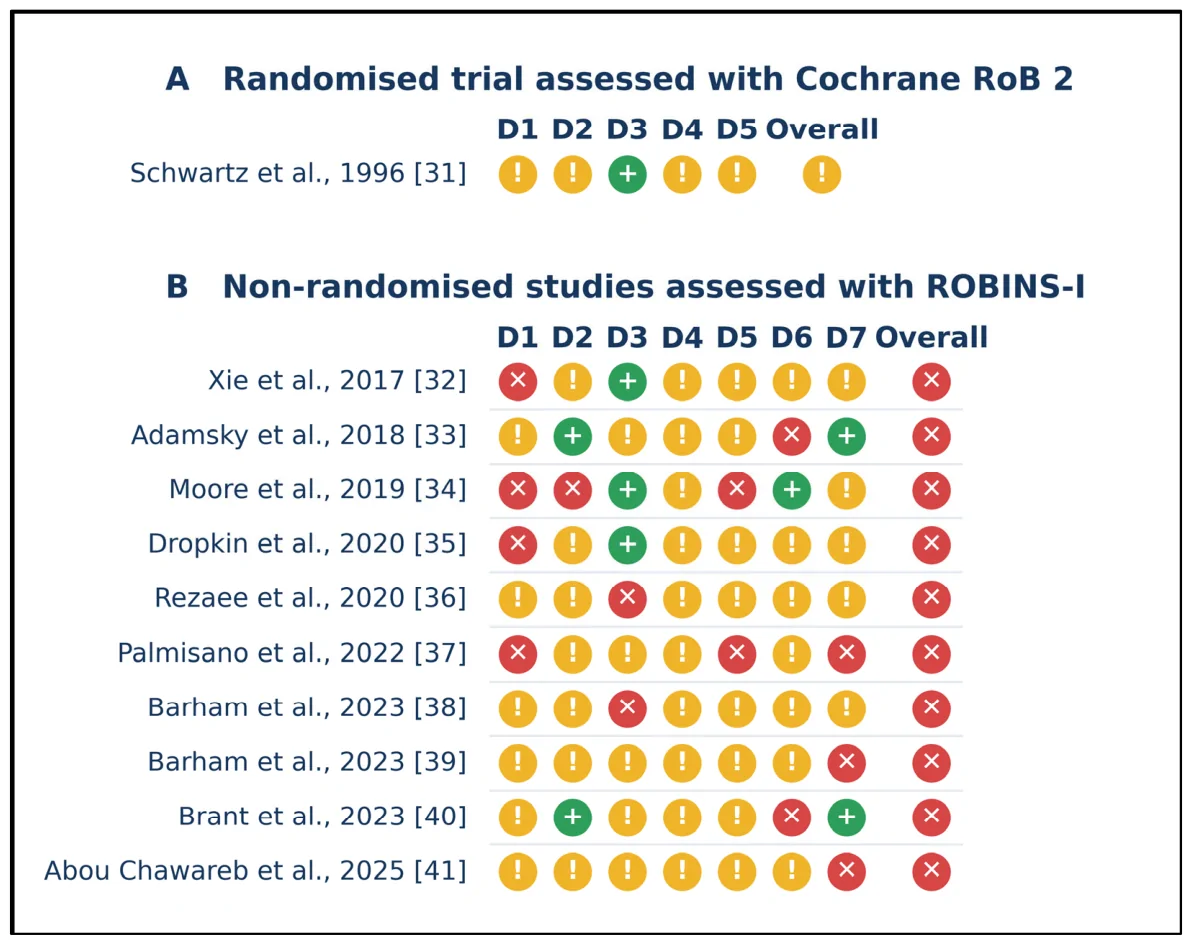
Risk-of-bias assessment. (**A**) The randomised trial [31] assessed with the revised Cochrane risk-of-bias tool (RoB 2). (**B**) The ten non-randomised studies [32,33,34,35,36,37,38,39,40,41] assessed with ROBINS-I. Judgements are those of the review authors. Green circles with a plus sign denote low risk of bias, amber circles with an exclamation mark denote moderate risk of bias or some concerns, and red circles with a cross denote serious risk of bias. RoB 2 domains: D1, randomisation process; D2, deviations from intended interventions; D3, missing outcome data; D4, measurement of the outcome; D5, selection of the reported result. ROBINS-I domains: D1, confounding; D2, selection of participants; D3, classification of interventions; D4, deviations from intended interventions; D5, missing data; D6, measurement of outcomes; D7, selection of the reported result.

**Table 1:** Characteristics of the included studies.

Study	Year	Country	Design	Data Source	N Analysed	Procedure	Population
Schwartz et al. [31]	1996	USA	RCT	Single centre	20	Primary/revision	Adults undergoing PP surgery
Xie et al. [32]	2017	USA	Retrospective cohort	Single centre	897	Primary and revision	Men undergoing IPP placement
Adamsky et al. [33]	2018	USA	Retrospective cohort	National claims database	10,847	IPP (mixed)	Men undergoing IPP placement
Moore et al. [34]	2019	USA	Retrospective cohort	Single centre	124	Implant, revision, salvage, explant	IPP, malleable PP or AUS
Dropkin et al. [35]	2020	USA	Retrospective cohort	Single centre	222	Primary	Men undergoing IPP insertion
Rezaee et al. [36]	2020	USA, Europe, South Korea	Retrospective cohort	Multicentre	804	Primary	Diabetic men undergoing PP surgery
Palmisano et al. [37]	2022	Spain	Retrospective cohort	Single centre	576	Primary and secondary	Malleable, two- or three-piece PP
Barham et al. [38]	2023	USA, Europe, Saudi Arabia, South Korea	Retrospective cohort	Multicentre	4161	Primary	Men undergoing IPP placement
Barham et al. [39]	2023	USA, Australia	Retrospective cohort	Multicentre	453	Revision (non-infected)	IPP revision for mechanical malfunction
Brant et al. [40]	2023	USA	Retrospective cohort	National hospital database	26,574	IPP (mixed)	Patients undergoing IPP surgery
Abou Chawareb et al. [41]	2025	USA, Europe, South Korea	Retrospective cohort	Multicentre	5261	Primary and revision	Men undergoing IPP surgery

Samples should not be summed because source cohorts overlap. AUS, artificial urinary sphincter; IPP, inflatable penile prosthesis; N, number of participants; PP, penile prosthesis; RCT, randomised controlled trial.

**Table 2:** Systemic antimicrobial exposures, comparators and pharmacological detail.

Study	Exposure Domain	Exposure	Comparator	Dose	Duration	Timing	Primary Outcome
Schwartz et al. [31]	Oral vs. parenteral regimen	Oral ofloxacin	IV gentamicin + cefazolin, then oral cephradine	NR	NR	Perioperative	Implant loss; reoperation; no infection occurred
Xie et al. [32]	Gentamicin dose	Gentamicin 5 mg/kg single dose	Gentamicin 80 mg 8-hourly	5 mg/kg vs. 80 mg (vancomycin 1 g both arms)	1 dose vs. 1 day	Preoperative	Device infection; causative organisms NR
Adamsky et al. [33]	Postoperative oral course	Oral antibiotic within 1 week	No postoperative oral antibiotic	NR	Mean 9.4 (SD 5.3) days	Postoperative	Explantation ≤ 90 days; causative organisms NR (administrative outcome)
Moore et al. [34]	Gentamicin dose	Weight-based gentamicin (5 mg/kg)	Standard gentamicin 80 mg	Median 5.06 vs. 1.07 mg/kg delivered	NR	Perioperative	AKI; device infection; causative organisms NR
Dropkin et al. [35]	Postoperative oral course	Discharge oral antibiotics (risk-stratified)	No discharge antibiotics	NR	NR	At discharge	Infection-related explantation; causative organisms NR
Rezaee et al. [36]	Regimen	AUA-recommended prophylaxis	Nonstandard prophylaxis	NR	NR	Perioperative	Device infection; explantation; causative organisms NR
Palmisano et al. [37]	Postoperative oral course	Discharge ciprofloxacin	Amoxicillin-clavulanate, other agent or none	NR	Inpatient course mean 1.4 days; discharge course NR	Discharge	Early PP infection; causative organisms NR
Barham et al. [38]	Regimen; antifungal; gentamicin dose	Vancomycin + gentamicin; antifungal (agent NR); weight-based gentamicin	Any other regimen; no antifungal; gentamicin 80 mg	Gentamicin weight-based or 80 mg; others NR	NR	Perioperative	Time to device infection; causative organisms NR
Barham et al. [39]	Regimen; antifungal	Vancomycin + gentamicin; antifungal (agent NR)	Absence of each (covariates)	NR	NR	Perioperative	Device infection; causative organisms NR
Brant et al. [40]	Regimen	Nonstandard or excess antibiotics	AUA-adherent regimen	NR (billing records)	NR	Perioperative encounter	Explantation ≤ 90 days; causative organisms NR (administrative outcome)
Abou Chawareb et al. [41]	Antifungal; oral course; IV duration	IV antifungal (agent NR); postoperative oral antibiotics; IV prophylaxis > 24 h	None; none; IV ≤ 24 h	NR	IV dichotomised at 24 h; oral NR	Perioperative	Device infection; causative organisms NR

Only systemic strategies are shown. Causative organisms NR indicates that the report did not present the causative microorganisms of the infections separately for the compared prophylaxis groups; agent NR indicates that the antifungal agent was not specified for the analysed exposure. AKI, acute kidney injury; AUA, American Urological Association; IV, intravenous; NR, not reported; PP, penile prosthesis; SD, standard deviation.

**Table 3:** Main comparative outcomes of the included studies.

Study	Comparison	Outcome	Exposed, Events/N (%)	Comparator, Events/N (%)	Crude *p*	Adjusted Estimate (95% CI)	Follow-Up
Schwartz et al. [31]	Oral ofloxacin vs. parenteral regimen	Implant loss or reoperation	0/NR (0)	0/NR (0)	NR	NR	Median 16 months (8–21)
Xie et al. [32]	Gentamicin 5 mg/kg vs. 80 mg 8-hourly	Device infection	3/407 (0.7)	6/490 (1.2)	0.057	NR	NR
		Gentamicin toxicity	0/407 (0)	NR	NR	NR	NR
Adamsky et al. [33]	Postoperative oral antibiotic vs. none	Explantation ≤ 90 days	NR (2.2)	NR (1.9)	0.18	OR 1.03 (1.002–1.05) per additional oral-antibiotic day	90 days
Moore et al. [34]	Weight-based vs. standard gentamicin	AKI	9/57 (15.8)	2/67 (3.0)	0.02	NR; crude OR 6.09 (1.26–29.49) a	7 days
		Device infection	3/56 (5.3)	3/58 (5.2)	1.00	NR	NR
Dropkin et al. [35]	Discharge antibiotics (two risk groups) vs. none	Infection-related explantation	NR (4); NR (5)	NR (0)	0.13	NR	Median 3.5–4.6 months
		Non-operative infectious complication	NR (2); NR (2)	NR (1)	0.83	NR	
Rezaee et al. [36]	AUA-recommended vs. nonstandard prophylaxis	Device infection	NR/391 (5.6)	NR/413 (1.9)	<0.01	OR 2.8 (1.1–7.3)	NR
		Explantation	NR/391 (8.3)	NR/413 (2.0)	<0.001	OR 3.6 (1.4–9.1)	
Palmisano et al. [37]	Discharge ciprofloxacin (infected vs. uninfected)	Early PP infection	5 (16.7) b	191 (39.0) b	0.014	Not entered into multivariable model	Mean 14.6 months (1–67)
Barham et al. [38]	Vancomycin + gentamicin vs. other regimen	Device infection	NR/2411 (1.0)	NR/1750 (1.2)	NR	HR 2.7 (1.4–5.4)	NR
	Antifungal (agent NR) vs. none	Device infection	NR	NR	NR	HR 0.08 (0.03–0.19)	
	Weight-based vs. 80 mg gentamicin	Device infection	NR	NR	NR	HR 2.9 (0.83–10)	
Barham et al. [39]	Vancomycin + gentamicin; antifungal (agent NR) (covariates)	Device infection	NR	NR	NR	Not associated after adjustment; estimates NR	Mean 13.0–18.3 months
Brant et al. [40]	Nonstandard vs. AUA-adherent regimen	Explantation ≤ 90 days	NR	NR	NR	OR 1.16 (0.78–1.71)	90 days
	Excess vs. AUA-adherent regimen	Explantation ≤ 90 days	NR	NR	NR	OR 0.91 (0.62–1.30)	
Abou Chawareb et al. [41]	IV antifungal (agent NR) vs. none	Device infection	NR	NR	<0.001	OR 0.22 (CI NR)	NR
	Postoperative oral antibiotics vs. none	Device infection	NR	NR	0.5	NR	
	IV prophylaxis > 24 h vs. ≤24 h	Device infection	NR	NR	0.2	NR	

Values are reported as published; missing denominators, confidence intervals and adverse-event data were not inferred. a Odds ratio calculated by the review authors from the reported event counts. b Proportion of infected and uninfected patients who received discharge ciprofloxacin (case–control orientation). Agent NR, antifungal agent not specified for the analysed exposure. AKI, acute kidney injury; AUA, American Urological Association; CI, confidence interval; HR, hazard ratio; IV, intravenous; N, number of participants; NR, not reported; OR, odds ratio; PP, penile prosthesis.

## Data Availability

All data analysed in this review were extracted from the published reports cited in the references.
